# Saudi Arabia’s costly war in Yemen: a neoclassical realist theory of overbalancing

**DOI:** 10.1177/00471178241231728

**Published:** 2024-02-13

**Authors:** Thomas Juneau

**Affiliations:** University of Ottawa

**Keywords:** balancing, neoclassical realism, overbalancing, Saudi Arabia, Yemen

## Abstract

Saudi Arabia faced multiple threats from Yemen in 2015: its southern neighbor had collapsed; a hostile sub-state actor, the Houthis, was entrenching itself along the border; and the presence of its rival Iran was growing. Responding was rational; it would have been sub-optimal for Riyadh to underbalance by doing little to counter the threat. Instead, however, Saudi Arabia overbalanced by launching a major air campaign and imposing a maritime and air blockade; as a result, it became bogged down in a costly war it cannot win. Why was this the case, and with what consequences? To answer this question, this article develops and applies a neoclassical realist theory of overbalancing. The first objective is nomothetic: to develop a theory of overbalancing, an important phenomenon neglected by the balancing literature. The second is empirical: to shed light on the Saudi decision to launch the war in Yemen.

Saudi Arabia faced multiple threats on its fragile southern border in 2015: Yemen had become a collapsed state in which a hostile sub-state actor, the Houthis, was entrenching itself and developing relations with Saudi Arabia’s regional rival, Iran. It was necessary for Saudi Arabia to mount a response given the threat emanating from Yemen; it would have been sub-optimal for Saudi Arabia to *under*balance by doing little or nothing. Instead, however, it *over*balanced against the threat by launching a major air campaign at the head of a coalition of 10 states and imposing a harsh maritime and air blockade. Saudi Arabia has since been stuck in a costly quagmire, with no realistic prospect for success.

To better understand the causes of Saudi Arabia’s sub-optimal response to the crisis in Yemen, this article develops and applies a neoclassical realist theory of overbalancing. This aims to fill a gap in the literature on balancing. Structural realism argues that faced with a threat, states are likely to balance; that is, they will generate, internally and externally, force to counter that threat. In practice, however, states often fail to respond in an optimal manner. When states fail to mount a sufficiently strong counter-response, they underbalance. The opposite sub-optimal response – overbalancing, or mounting a disproportionate and unnecessarily strong counter-response – has been neglected by scholars.

This article has twin objectives. The first is nomothetic: to contribute to the development of a theory of overbalancing, an important phenomenon neglected by the balancing literature. The second is empirical: to shed light on the Saudi decision to launch the war in Yemen.^1^ The article starts by building a theory of why states deviate from optimal balancing behavior, with the focus on one particular case of sub-optimal foreign policy, overbalancing. It then applies this framework to Saudi Arabia’s intervention in Yemen.

## Balancing and overbalancing

The balancing proposition is central to International Relations theory: faced with an aggregation of power, a state – because it inhabits an anarchic world where self-help is the only road to survival – will itself generate power internally by building arms or externally by forming alliances, or both.^2^ By balancing, the state’s objective is to ‘amass military might so as to deter another’s aggression or prevail in a conflict should deterrence fail’.^3^ The proposition is often viewed as descriptive: balancing is how states typically behave: according to Waltz, ‘faced with unbalanced power states try to increase their own strength or they ally with others to bring the international distribution of power into balance’.^4^

In practice, however, states regularly fail to balance. Schweller labels this as underbalancing, which ‘occurs when the state does not balance or does so inefficiently’.^5^ Scholars have noted that underbalancing is frequent in the Middle East: Gause, notably, argues that the inability of Saudi Arabia, Turkey, and Egypt to counter-balance Iranian gains since 2003 amounts to underbalancing. In his view, conventional balance-of-power logic fails to offer a comprehensive explanation; alignment patterns in the region are driven more by ideological compatibility and regime similarity than by straightforward power considerations.^6^ There is very little in the literature, however, on the opposite phenomenon, overbalancing. In his work on underbalancing, Schweller only mentions it briefly.^7^ Important works on balancing make little or no reference to it. Edited volumes by Vasquez and Elman and by Paul, Wirtz, and Fortmann notably have no index entry for overbalancing.^8^

There is, as such, a gap in the literature. What is overbalancing? It emerges when a state overestimates a threat and mounts a disproportionately strong response, or when it correctly perceives a threat but its domestic politics push it toward an exaggerated response. I thus define overbalancing as such: a state overbalances when it faces a threat calling for a balancing response but, because foreign policymaking is hijacked by domestic political processes, the amount of counterforce it generates (domestically through internal balancing and externally through alliances) is greater than what the threat optimally calls for. The response is therefore less likely to allow the state to maximize its security as an optimal balancing response would.

The phenomenon of overbalancing corresponds to a set of cases in which states fail to balance. As such, other theories of balancing – which typically explain how and why states balance – cannot explain cases of sub-optimal behavior whereby states fail to respond optimally. Walt’s balance-of-threat theory, which argues that states balance against threats rather than against power, would arguably have been appropriate to explain its actions in Yemen had Saudi Arabia reacted proportionately to the threat posed by the Houthis.^9^ But because it reacted disproportionately, a theory of mistakes is necessary. Similarly, omnibalancing explains *how* a state responds to threats; it is not a theory of mistakes.

A theory of overbalancing must also answer a fundamental question: is failure a prerequisite? That is, can the overbalancing label be attached to a state’s actions *only* if it fails to achieve ideal objectives (i.e. maximizing its security by balancing optimally against the threat)? The answer is no, or at least, not necessarily: overbalancing can be successful, in the sense that a state can engage in this type of sub-optimal behavior and still maximize its security. But this would still be a costly success: more resources than necessary would have been used to reach optimal objectives, also implying that these resources would then not be put to more productive use elsewhere. This has to be further nuanced. Failure, first, is not certain but is more likely when a state overbalances, since its maximalist objectives will exceed what is feasible or ideal. More precisely, the more the state overbalances, the more the costs are likely to be high.

The concepts of under- and overbalancing are similar to omnibalancing, but also different in key respects. Omnibalancing refers to the reality that governing regimes in Global South states often face both domestical and international threats. In this context, they seek alignment with external powers that can help them deal with these multiple fronts.^10^ The concept has been applied in some of the best analyses of the foreign policy of Middle East states in recent decades, including Saudi Arabia. Mason and Nonneman, in particular, have argued that Saudi foreign policy displays patterns of omnibalancing, as Riyadh has traditionally sought to ally with external states – chiefly the United States – to protect itself against domestic (e.g. Sunni radicals), regional (Iran today, and before that radical Arab states), and international (the Soviet Union) threats.^11^

A theory of overbalancing is also distinct from other theoretical frameworks focusing on sub-optimal behavior. Overbalancing, first, is distinct from what Snyder defines as imperial overexpansion. He argues that elite fragmentation can lead to grand strategies of pathological overexpansion. Cartelized regimes representing a narrow set of actors with parochial interests tend to favor militarist policies and stimulate hyper-nationalism, leading to self-defeating expansionism.^12^ As Schweller argues, such overexpansion is driven by greed; it is an attempt at power-maximization.^13^ Overbalancing, on the other hand, is an extreme form of balancing; it is an attempt at security-maximizing.

Overbalancing also overlaps with but differs from another related phenomenon, misperception. Waltz’s structural realism assumes that states perceive more or less automatically and correctly the intensity and imminence of threats. Others, especially Jervis, challenge this premise, arguing that decision-makers often misperceive their environment.^14^ Neoclassical realists agree that misperceptions frequently lead to maladjusted responses. Wohlforth, for example, argues that divergent assessments of shifts in the distribution of power between the United States and the Soviet Union regularly led to crises.^15^ As discussed below, misperceptions (more specifically, inflated perceptions) are crucial to a theory of overbalancing. But central to neoclassical realism is the desire to gain specificity and accuracy, albeit at the cost of parsimony. The perceptions variable carries some causal weight, but it is too crude to explain overbalancing on its own: a state can misperceive by inflating a threat but still avoid overbalancing if other variables at the domestic level constrain its ability to act on the basis of those inflated perceptions.

## A neoclassical realist theory of overbalancing

For neoclassical realists, a state’s relative power is the chief determinant of its foreign policy.^16^ This is what makes them realists. They also believe that frameworks based solely on structural factors explain the broad context in which a state operates but are underspecified. Neoclassical realism thus further posits that domestic factors act as transmission belts filtering systemic pressures, converting them into actual foreign policy.^17^ Since Rose proposed the term in 1998, the neoclassical realist research program has given rise to three approaches.^18^ Type I proposes a theory of mistakes: it explains why states adopt sub-optimal foreign policies that fail to respond to systemic imperatives.^19^ Type II expands its scope beyond anomalies to a broader range of foreign policy choice and grand strategic adjustment. In this case, the independent variable (power) explains the broad parameters of foreign policy, while intervening domestic variables explain the residual variance. More recently, Ripsman, Taliaferro, and Lobell, who suggested this typology, broadened the scope of the neoclassical realist research program. Whereas Types I and II focus on foreign policy analysis, they argue that over time, individual foreign policy choices affect international outcomes and, therefore, systemic structure itself. This is what they label Type III, or neoclassical realism as a theory of international politics.^20^

Type I neoclassical realism provides the ideal framework to explain overbalancing, a textbook case of sub-optimal foreign policy. A theory of mistakes starts by differentiating ideal foreign policy, which broadly follows the pressures and incentives of the system, from actual foreign policy, which deviates from this ideal.^21^ The ideal version allows a state to achieve its objectives; in this case, that implies maximizing its security by balancing proportionally against a threat. In the sub-optimal scenario, domestic variables hijack foreign policy and steer it away from optimal outcomes, leading the state to suffer costs. Here, instead of a proportional balancing response, the state generates disproportionate counterforce against a threat.

A neoclassical realist theory of overbalancing thus consists of four stages: the definition of ideal foreign policy, and then the three stages in the causal chain explaining actual foreign policy: the independent variable (power, shaping the parameters of the country’s response), the intervening variables (how domestic processes hijack foreign policy), and the dependent variable (overbalancing).

### Ideal foreign policy

The first step in building a neoclassical realist theory of mistakes is to conceptualize the baseline, ideal foreign policy that states *should* follow. For a theory of overbalancing, this refers to the optimal balancing response, or the proper amount of power it should generate to counter the threat. This is what Lobell labels as appropriate balancing: the ‘proportionate arms buildup or the formation of an offsetting alliance against the threatening element(s) of power’; it is against this ideal that the sub-optimal, actual response can be assessed.^22^

This baseline calls for states to respond to systemic pressures independently of domestic constraints. The challenge is that there is no consensual structural realist prescription. In different contexts, different strands within structural realism offer a range of options. For a theory of overbalancing, it is logical to build this baseline on the prescriptions of defensive realism, and not those of offensive or other variants of structural realism. The objective of balancing is for a state, faced with a threat, to maximize its security, not its power. Defensive realism – which starts with the assumption that states are security-maximizers^23^ – thus provides the most appropriate framework.

Defensive realism argues that states should focus on protecting what they have as opposed to covet what they do not. They should refrain from expansionist policies and instead embrace prudence; they should exercise restraint, especially in the face of non-existential threats. Defensive realists do not view restraint as an end in itself, but rather as a means to achieve security.^24^ Force, additionally, is inefficient as a tool of state-building: defensive realists are skeptical of externally imposed social engineering. When force is used, it should be as a last resort and follow an austere cost-benefit calculus. What would be a defensive realist prescription for an ideal balancing response? Because they are skeptical of the effectiveness of the large-scale use of force, defensive realists prefer policies focused on deterrence, containment, and homeland defense.^25^ An optimal balancing strategy should pursue these objectives through both internal (generating its own force) and external (building alliances) means.

### Independent variable

For neoclassical realists, a state’s relative position in the international system is the chief determinant of its foreign policy. Power is a permissive cause: it shapes foreign policy but does not precisely determine it. It shapes the broad parameters in which a state can act, but within these, there remains significant margin of maneuver.^26^ There are two steps to understand how power shapes the overbalancer’s foreign policy. The first is to assess its relative place in the international or regional balance of power. The second is to assess the distribution of power specific to the dyad between the overbalancer and the threat itself. Here, neoclassical realism must identify whether specific configurations are more or less likely to favor the emergence of overbalancing. A power vacuum, in particular, can be a necessary (but far from sufficient) condition: the sudden opening of a window of opportunity, if for example a rival collapses, can lead to the disappearance of constraints on a state’s ability to assertively project power. This is not a rigid causal relationship, however; domestic political factors can further steer the state toward overbalancing, or constrain it.

### Intervening variables

Intervening variables explain the conversion from the possible to the actual.^27^ They are domestic processes explaining how a state’s power, the usable assets it can bring to the table, is translated into action, or how the pressures and incentives shaped by a state’s international position are translated into actual choice. The independent variable, in other words, provides the permissive context, and the intervening ones the proximate causes. This is an important distinction: states faced with a similar international environment may come up with different responses because of their different internal characteristics. Relative position is a necessary but insufficient condition to explain state behavior.

A state’s place in the international distribution of power shapes the range of its possible courses of action; specific choices within this structurally determined bandwidth are then explained downstream by the additional causal impact of intervening variables. Realists have suggested a comparable role for domestic politics in the past, without systematizing it as those of the neoclassical strand have recently attempted. Morgenthau, notably, left room for domestic politics to specify the relationship between power and interests: ‘contingent elements of personality, prejudice, and subjective preference, and of all the weaknesses of intellect and will which flesh is heir to, are bound to deflect foreign policy from their rational course’.^28^ Similarly, in the very last sentence to *Man, the State, and War*, Waltz foreshadowed the logic of neoclassical realism: ‘The third image describes the framework of world politics, but without the first and second images there can be no knowledge of the forces that determine policy’.^29^ Intervening variables thus have a constricting effect, narrowing the range of choice by removing from consideration options that are in theory available based on the state’s power.^30^ Ideas, for example, or the constraints imposed by the domestic coalition that keeps a government in power, can lead to the elimination of options possible solely on the basis of the state’s capabilities.^31^

Neoclassical realism’s incorporation of domestic political processes as intervening variables into its causal chain represents one of its most important innovations; it is also one of its most controversial. Legro and Moravcsik were the first and most prominent among the critics, accusing neoclassical realism of stretching paradigmatic boundaries beyond recognition, costing it much of its coherence and distinctiveness. Narizny, for his part, argues that its ad hoc integration of a seemingly endless menu of domestic variables makes it fundamentally flawed.^32^ These are valid criticisms: it is undeniable that there is scope for neoclassical realism to increase its rigor. There remains an especially glaring need for neoclassical realists to better define intervening variables and better explain why they choose which intervening variables, in which circumstances, and with which causal effect. That said, they have made progress in the past decade: while neoclassical realists continue to produce a growing array of innovative case studies, they have also expanded the scope of the research agenda and strengthened its foundations.^33^ As discussed below, scholars such as Foulon, Götz, and Meibauer have made significant advances at this and other levels.

Neoclassical realists have offered various typologies of intervening variables. Foulon, for example, identifies four categories: policymakers’ cognitive filters, domestic ideology, interest groups, and state extractive capabilities.^34^ Ripsman, Taliaferro, and Lobell, for their part, suggest four similar categories: leader images, strategic culture, state-society-relations, and domestic institutions.^35^

Among those, two particular intervening variables explain the deviation toward overbalancing: leader images and resource mobilization (itself a subset of state-society relations). To understand why it is necessary to have these two and not others, and how they combine to complete the causal chain, it is useful to introduce Götz’s framework differentiating types of intervening variables. The first, leader images, corresponds to what Götz labels a complementary factor: it affects the ‘nature, timing, and duration’ of the overbalancer’s response and helps explain the ‘exact form or manner in which states respond to systemic stimuli’.^36^ The causal impact of the second, resource mobilization, corresponds to a moderating factor: it conditions the government’s ability to respond adequately or not to systemic pressures. Such variables ‘influence the strength of the relationship between presumed cause and effect’.^37^ In this case, the stronger the state’s ability to mobilize resources, the stronger the government’s ability to respond will be. The combination of leader images and resource mobilization, a complementary and a moderating factor, explains the choice of these two variables; as discussed above, it is essential for neoclassical realism to move away from the ad hoc selection of such variables and to be able to justify their selection on the basis of more rigorous criteria. Here, it makes sense to combine a complementary factor, which shapes the nature of the response, with a moderating factor, which shapes its strength. Together, they explain why, given a certain international environment, a state veers toward overbalancing.

The first intervening variable, leader images, includes the set of ‘core values, beliefs, and images that guide’ how leaders understand the world. Such cognitive biases and preferences shape how leaders assess their options.^38^ Leaders’ images push them to dismiss some facts and emphasize others, and interpret specific pressures and incentives more or less favorably. This nudges policymakers to favor certain options and reject others on the basis of pre-existing belief systems, narrowing the range of conceivable options.^39^ According to Meibauer, they function as cognitive devices or mental shortcuts that fill knowledge gaps and help to make sense of complex situations in contexts where information is incomplete and uncertain. It points decision-makers to view some information as more or less relevant.^40^

Leader images include individual cognitive characteristics of the state’s leadership, what Ripsman, Taliaferro, and Lobell refer to as the foreign policy executive, those who ‘sit at the helm of the state’.^41^ If the leadership is ambitious and supports an aggressive foreign policy seeking to position the state as a dominant power, it will read the aggregation of power it faces differently than if another state, in a similar position, is led by cautious, risk-averse individuals. Byman and Pollack, in particular, have shown how unusually strong leaders can have a disproportionate impact on foreign policy. In those cases, these leaders’ preferences become a key variable shaping choice.^42^

Leader images impact how leaders assess the aggregation of power their country faces: whether it is a threat or not, and if yes, how significant they believe that threat to be. This is a variable commonly used in neoclassical realism. In addition to Wohlforth’s work mentioned above, He has explained how the pattern of competition and cooperation between the United States and China from 1949 to 2015 has been predominantly shaped by how leaders of each country perceived how the other threatened their own security and economic interests.^43^ This step plays a hinge role in the causal chain: if leaders do not perceive the aggregation of power as a threat, then we are not in the realm of balancing. If they do, then the mechanism for the launching of a balancing response can be initiated. If they do perceive it as a threat, the next key question is whether they evaluate it accurately. If their perceptions of the intensity and imminence of the threat are accurate, this tends to nudge the state toward an appropriate response. Underestimating the threat tends to nudge it away from the ideal baseline and toward underbalancing. An inflated threat perception, on the other hand, can be a driver of overbalancing: the more a state assesses a threat to be greater than it is in reality, the more it will be likely to mount an exaggerated response.

This is still insufficient to explain the outcome on its own: a state can overperceive a threat but still balance proportionately if the next steps in the causal chain bring the response back to the optimal level. The state may perceive an inflated threat but if it does not have the ability to mobilize significant resources, it will be steered away from overbalancing. As Schweller argued, the ability for the state to extract resources from society and mobilize power is key to the operation of the balancing mechanism.^44^ A given state may face an aggregation of power and perceive an inflated threat; three scenarios are then possible. If it is unable to mobilize resources, it will fail to balance proportionately; it will underbalance. If the government faces some constraints – enough that it cannot mobilize a disproportionately high amount of counterforce, but not to the extent that it will fail to mobilize – then it is more likely to generate a response more or less proportional to the threat. But if the state faces few constraints on its ability to mobilize power, then it becomes more likely to generate an unnecessarily strong counterforce; overbalancing is more likely.

More specifically, a united regime in which power is concentrated in the hands of a smaller number of like-minded elites will mobilize resources more effectively; greater elite cohesion, leads to a less fragmented system and a faster ability to make decisions.^45^ On the other hand, a fragmented regime where decisions follow extensive consultation with multiple power centers is likelier to lead to slower decision-making.^46^ It is then difficult to rapidly mobilize resources to act swiftly on the international stage, making underbalancing more likely. The consolidation of power in a smaller number of more like-minded elites, on the other hand, makes the ability to decide, mobilize resources, and act more likely. With fewer checks, restraints on bad decisions are removed; this opens the door for the mounting of an exaggerated response.

**Figure 1.**
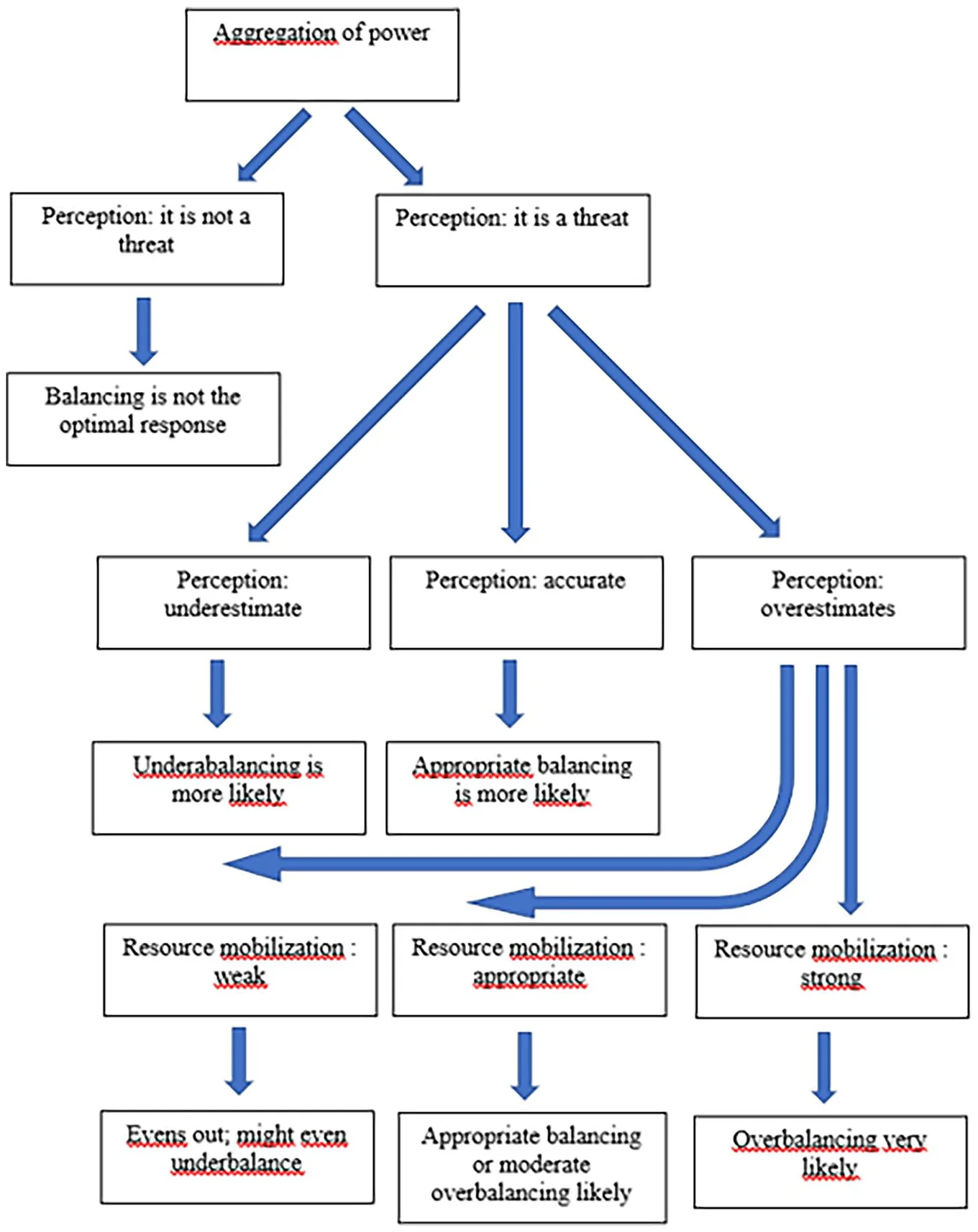
Pathways to balancing and overbalancing.

Taken together, these two intervening variables, in combination with the dependent variable, offer a causal chain explaining the emergence of overbalancing (see Figure 1). At the international level, specific configurations of the relative distribution of power, such as the sudden opening of a window of opportunity, provide the permissive context that can open the possibility of a state overbalancing. The state’s leadership then assesses a concentration of power to be more threatening than it actually is. Finally, the state has the ability to mobilize a significant amount of power; it is not, for example, weak or paralyzed and thus unable to extract resources from society. This last step is essential: it is the generation of this counterforce domestically (by building military power) and externally (gathering allies) that leads to the actual overbalancing response.

The result is, admittedly, a complex causal chain incorporating an independent variable and two intervening variables. Those who prefer the sparser theories of Waltzian realism will disapprove of this lack of parsimony. Neoclassical realists, however, accept the costs that come with decreased generalizability and emphasize gains in accuracy and specificity. This approach to theory-building is also reflected in the operationalization of the intervening variables. They do not take a ‘yes’ or ‘no’ value, but rather values along a continuum, from low to high or weak to strong. The more these variables take on a high value (threat perceptions are inflated, the state has a strong ability to mobilize resources), the more the state is likely to overbalance; on the other hand, if most variables take on a roughly middling value, one would expect a response closer to optimal balancing. This embrace of complexity is consistent with the soft-positivist epistemology of neoclassical realism.^47^

### Dependent variable

A state overbalances when it generates and commits too many resources against a threat; balancing is warranted, but the actual response is inflated and disproportional to the threat. The state deviates above the ideal baseline (a balancing response proportional to the threat), generating too much counterforce. This is a sub-optimal outcome which leads to negative consequences.

**Figure 2.**
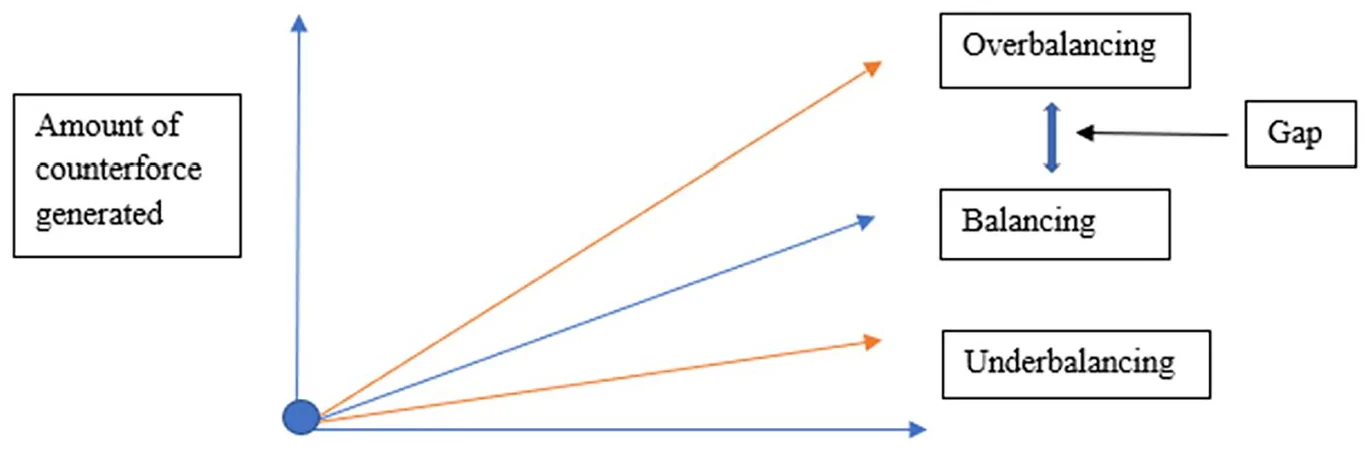
The gap between balancing and overbalancing responses.

The crucial first step here is to demonstrate empirically the existence of a gap between ideal foreign policy and the actual outcome. It is only if the outcome is above the baseline that there is an overbalancing response (Figure 2). This is a key step: there is no overbalancing if the existence of this gap cannot be demonstrated. The empirical analysis must also show that this outcome is specifically the result of the operation of the causal chain.

A necessary element at this stage is to demonstrate how the outcome – overbalancing – is distinct from other, similar but non-balancing types of responses such as overexpansion. The analysis must include the empirical demonstration that the overbalancing outcome is a subset of a balancing response. The account of actual foreign policy must provide a detailed account of how the overbalancer generated too much counterforce both internally and externally, and used it to counter the perceived threat. The final step is then to analyze the negative consequences of overbalancing. Pursuing a sub-optimal outcome is costly: deviating from ideal foreign policy prevents the state from maximizing its security.

## Overbalancing: Saudi Arabia’s intervention in Yemen

Insecurity emanating from Yemen threatens Saudi Arabia; it is inevitable for Riyadh to be concerned with developments in its southern neighbor.^48^ This insecurity can take many forms: an aggregation of power along Saudi Arabia’s vulnerable southern border (if a strong and hostile regime takes over in Sana’a or a rival power establishes a foothold) or, conversely, a power vacuum as a result of state fragility or collapse (which can lead to war or terrorist groups building safe havens).^49^ At the time of unification between North and South Yemen in 1990, Saudi Arabia feared that a unified Yemen could potentially rival Saudi power on the Arabian Peninsula. Subsequent instability, however, made this scenario unlikely as Yemen remained fragile after unification.^50^ There were multiple drivers of instability: many in the south harbored grievances toward the north^51^; the Houthis, who emerged in the 1990s by tapping resentment in the northwest because of neglect by the center and attempts to spread Salafi Islam in a predominantly Zaydi Shia area, held six rounds of fighting with the central government starting in 2004^52^; and the country emerged as a stronghold for Al-Qaeda, which established its local franchise, Al-Qaeda in the Arabian Peninsula (AQAP), in 2009.

Yemen was thus a fragile state when the Arab uprisings reached it in 2011. After months of protests and intra-elite fighting, President Ali Abdullah Saleh resigned, paving the way for his vice-president, Abdrabbu Mansour Hadi, to be elected president in 2012. Under a deal backed by Saudi Arabia and the United States and supported by the UN, Hadi was to lead a national dialogue and build consensus toward a new constitution. The process collapsed, however, and the country fell into civil war in 2014 when the Houthis, increasingly close to Iran and suddenly allied with Saleh (who had maintained significant loyalty among the security forces), seized Sana’a.^53^ The Houthis continued advancing southward, launched maneuvers close to the Saudi border, and announced an air bridge connecting Tehran and Sana’a. With his government on the verge of collapse, Hadi requested assistance from Saudi Arabia in March 2015.

### Competing explanations

Available accounts of the Saudi decision to intervene in Yemen often explain the war through the prism of the role of leaders, specifically the rise of the ambitious Saudi Crown Prince Mohammed bin Salman (MbS) after his father, Salman, acceded to the throne in January 2015. According to this view, MbS rapidly took control of the levers of power and jettisoned decades of cautious and risk-averse foreign policy, seeking to implement his ambition of positioning Saudi Arabia as a more assertive regional power.^54^ Comparing the rise of MbS in Saudi Arabia and Mohammed bin Zayed in the United Arab Emirates, for example, Davidson focuses on the role of each strongman in centralizing power and implementing their own vision.^55^ Similarly, Al-Rasheed focuses on MbS’s personality to explain the sudden aggressiveness of Saudi foreign policy.^56^

Moving beyond personality-based accounts, Darwich explains the aggressiveness of Saudi foreign policy since 2011, illustrated by the intervention in Yemen, by the Kingdom’s quest for recognition of its status as a regional power.^57^ Similarly, she explains the persistence of the costly Saudi commitment to a failed war by building on the literature on status.^58^ Others argue that the main driver of Saudi foreign policy since 2011 has been a growing perception of threats to regime security driven by fear of the protests that swept the region in 2011,^59^ acting as a key driver of the interventions in Bahrain in 2011 and in Yemen after 2015.^60^ Orakby, for his part, focuses on Riyadh’s perceptions of the external threat posed by Houthi expansion on its southern border.^61^

Still others shift their analysis to the regional level, framing the war as one element in the confrontation between Iran and Saudi Arabia, the two dominant regional powers across the Persian Gulf. In the zero-sum game they have been engaged in, gains by one are perceived automatically by the other as a loss, pushing them in a spiral of competition, of which Yemen has been the victim.^62^ Some frame their analysis on the basis of sectarianism, explaining the war through the prism of the Sunni-Shia struggle for regional influence between the two regional powers.^63^ Going back to the events of 2011, Al-Rasheed argues that sectarianism was the ‘avenue’ for Saudi Arabia to oppose the wave of Arab uprisings.^64^ Another promising stream in the literature analyzing Saudi foreign policy argues that it has become, under King Salman and MbS, characterized by hedging, or an effort to diversify partnerships beyond the traditional one with the United States. For Demmelhuber, notably, growing Saudi assertiveness has also featured growing independence from Washington.^65^

These accounts partially explain the Saudi decision to launch the intervention, but each also suffers from various flaws, notably their focus on a single variable. Emphasis on the role of personalities and threat perceptions, for example, rightly highlight important domestic processes in Saudi Arabia, but it misses the larger context: structural variables shape the broader parameters in which personalities and perceptions impact choice. A state can see a shift in its perceptions of the evolving balance of power or the emergence of a strong leader, for example, but a corresponding shift in foreign policy is not automatic: structural factors can act as constraints preventing an ambitious leader to pursue his or her objectives. Accounts focusing only on structural variables (the balance of power between Saudi Arabia and Iran), for their part, appropriately frame the parameters of the Saudi decision but lack the specificity that would come from combining this structural variable with more granular domestic factors. Similarly, accounts focusing on sectarianism have come under criticism; Saudi Arabia and Iran do mobilize sectarian identities in the pursuit of their goals, but this is in the realm of tactics, not drivers of foreign policy.^66^ The Saudi response, moreover, has been disproportional and costly. As such, a theory of mistakes is necessary to explain the emergence of these negative consequences. Such a theory should incorporate multiple variables: power, leader images, and resource mobilization matter, but each explains only aspects of the decision; none provides a comprehensive account alone.

Single-variable explanations, in other words, are incomplete and too parsimonious. In one notable exception, Ehteshami offers a more comprehensive account of the growing assertiveness of Saudi foreign policy, combining explanatory factors at the regional (the evolving balance of power) and domestic (policy construction by elites) levels.^67^ Similarly, Sunik argues that regional power shifts opened a window of opportunity for Saudi Arabia’s growing assertiveness. She then combines this structural element with the symbolic functions of autocratic institutions to explain more precisely the shape of the new Saudi foreign policy and its bid for regional leadership.^68^ What is missing in both cases, however, is the sub-optimality dimension. A neoclassical realist theory of overbalancing provides such an alternative. Like Ehteshami and Sunik, it sacrifices parsimony in favor of accuracy and specificity, while also providing a more comprehensive framework to explain foreign policy mistakes.

### The ideal baseline

As the situation in Yemen in early 2015 threatened its security, it was inevitable that Riyadh would respond. The Houthis, hostile to Saudi Arabia, were rapidly expanding the territory under their control and had seized the capital Sana’a the previous year. They were growing in strength, in part because of their absorption, by cajoling or coercing them, of units from the national army. It was also becoming clear that Iran, Saudi Arabia’s chief regional rival, was ramping up its support – marginal until then – for the Houthis.^69^ Mounting a balancing response proportional to the threat would have been the optimal response.

This ideal response would have aimed to avoid entanglement in an unwinnable war. It would have been premised on fortifying its southern border to deter Houthi incursions, supporting anti-Houthi forces, and pushing for a political settlement. The choice of local partners would have been crucial: by tying its fortunes to Hadi, a corrupt and ineffective leader, Saudi Arabia severely damaged its ability to accomplish realistic objectives. This optimal approach could have included limited airstrikes to damage Houthi capabilities and try to compel them to negotiate. Saudi Arabia also should have built a regional coalition to contain and deter the Houthis. The pooling of resources that a broad and engaged coalition offers would have added an important, external element to Saudi balancing.

Not sufficiently intervening – underbalancing – would certainly have been sub-optimal. Absent Saudi support, the Hadi government would have likely collapsed, or at least it would have become even weaker, leading to the further empowerment of the Houthis. This would have allowed the Houthis to entrench their position on the south-west corner of the Arabian Peninsula. Yet Saudi Arabia’s actual response – a prolonged, large-scale air campaign at the head of a coalition of 10 states and a maritime and air blockade, which has led Saudi Arabia to become bogged down in a highly costly war with no clear path to victory – has been out of proportion to the threat. There is, as such, a gap between ideal and actual policy: Saudi Arabia has overbalanced in response to the threat. Why, and with what consequences?

### Independent variable

The independent variable – Saudi Arabia’s place in the international distribution of power – is the permissive condition setting the context for its overbalancing response. Two aspects of the regional balance of power matter: Saudi-Iranian competition in the Gulf and a power vacuum toward Saudi Arabia’s south as a result of the collapse of the Yemeni government.

The balance of power in the Persian Gulf features two dominant players, Iran and Saudi Arabia (especially since Iraq’s collapse in 2003).^70^ Irrespective of the worldview of the rulers in each, by dint of the two countries’ geographical, economic, religious, and human assets, their leaders tend to see their own country as a regional power. This bipolar sub-systemic distribution of power implies that strategic competition is probable, though it does not imply automatically high levels of tension. Indeed, certain eras such as the 1970s witnessed somewhat cooperative relations as both shared comparable visions of regional order; yet even then, suspicion and mistrust remained high.^71^ Saudi Arabia and Iran prefer to avoid direct confrontation: Riyadh realizes that despite its inferiority in conventional military terms, Tehran could inflict significant damage through its missile and drone programs and its support for armed non-state actors surrounding Saudi Arabia, while Iran is deterred by the strong regional American presence. Instead, they confront each other indirectly by supporting rival militant groups in weak or collapsed states throughout the region. This has been the case, notably, in Lebanon, Syria, and Iraq. In 2014/15, as Houthi power and ties to Iran expanded and as the Saudi-backed internationally recognized government weakened, Yemen emerged as another theater in this region-wide confrontation.^72^

To this Saudi-Iranian dyad must be added the specific context of the Saudi-Yemeni balance of power. Saudi Arabia is the dominant power on the Arabian Peninsula, while Yemen is a historically weak state that by 2015 had collapsed. A stronger power facing a vacuum is subject to intense pressures and incentives to expand its interests. Systemically, this acts as a permissive condition encouraging overbalancing, but an ambiguous one: the message is to exploit the vacuum, but how to do so precisely is unclear. There is, in other words, significant scope for domestic factors to have a strong causal impact on foreign policy.

### Intervening variables

In sum, the regional context – the independent variable – features elements that shape a mildly strong permissive context for overbalancing, but it does not provide immediate causes; structure can potentially contribute to an overbalancing response, but on its own it is not sufficient to explain it. It represents the broad framework shaping the Saudi margin of maneuver. Had Riyadh’s threat perceptions been less inflated or its ability to mobilize resources been more constrained, overbalancing would have been less likely, and perhaps impossible. Instead, inside the range of options shaped by power, domestic political processes further nudged the country toward overbalancing.

Leader images, the first intervening variable, consists of the cognitive biases and preferences that shape how leaders view the world and assess their options. Ambitious leaders who are more risk tolerant and do not fear confrontation, in particular, are more likely to overbalance, while more modest, risk-averse, and confrontation–shy leaders are more likely to underbalance. To operationalize this variable, two steps are thus necessary: to understand the worldview of the leadership, and then the implications for its perception of the threat the state faces. The second step is crucial: in contexts of crisis decision-making, leaders – pressed for time and overwhelmed by contradictory information – can be especially likely to misread opponents’ intentions and capabilities.

The emergence of MbS has clearly had a strong impact on Saudi foreign policy. MbS has been critical of what he views as Saudi Arabia’s traditionally cautious and reactive foreign policy. He favors instead more proactive participation in regional affairs, less based on seeking consensus domestically and among Arab states and more based on the confident projection of Saudi power. He also favors an assertive Saudi nationalism; as one interviewee explained, he promotes a ‘Saudi Arabia first’ foreign policy. He is also far more tolerant of risk than his risk-averse predecessors. A leaked memo by the German intelligence agency, for example, referred to him as a ‘political gambler’.^73^ MbS, moreover, favors a bolder confrontational stance toward Iran. In his view, as confirmed by multiple interviews, the previous King, Abdullah, was mistakenly trying to manage tensions with Iran and ended up constantly reacting defensively to, and thereby facilitating, Iranian advances. As one interviewee explained, MbS ‘felt that Saudi had punched below its weight. . . (as a result), he threw caution to the wind’.

MbS’s worldview had concrete implications that contributed to shaping the Saudi response to the situation in Yemen, especially through Saudi perceptions of American retrenchment from the Middle East and of the threat posed by Iran. Riyadh, first, believed that the Obama Administration was engineering a retrenchment of American power from the Middle East; this perception was exaggerated, but as virtually all interviews confirmed, it was a dominant view in Riyadh, and one that stoked significant concern. The perception crystallized with what Saudi Arabia viewed as the United States jettisoning Egyptian President Hosni Mubarak in 2011 and, more generally, as the Obama Administration tolerating, even encouraging, the protests that erupted region-wide in late 2010. As interviewees emphasized, Saudi Arabia did not feel that the United States supported its efforts to roll back the revolutionary tide, as witnessed by American criticism of the Saudi intervention to protect the Bahraini monarchy. Riyadh also grew frustrated at what it believed was American willingness to accept the rise of the Muslim Brotherhood in post-uprising Egypt and elsewhere. President Obama’s restraint in Syria further angered the Saudis, who hoped for greater American efforts to topple the Assad regime^74^; in the words of one interviewee, it made Saudi Arabia ‘livid’. It was especially fearful that Washington would tilt toward Iran, viewing the 2015 nuclear deal as a betrayal; one Saudi interviewee referred to the U.S. and Iran as ‘conspiring’ against Saudi Arabia.^75^ Finally, multiple interviews confirmed that the Obama administration’s coolness toward the intervention in Yemen amplified this perception: the US chose to support the intervention, in part to ensure that Saudi Arabia would not oppose ongoing nuclear negotiations with Iran, but it was not enthusiastic about it.

Overall, multiple interviewees emphasized the dominant perception in Riyadh of America’s ‘weakness’ and of its ‘unwillingness to intervene’. According to this perception, this gave space to Saudi rivals, especially Iran, to expand in the vacuum. Many interviewees recognized that this perception was inflated – the United States was not withdrawing from the region, but was merely exercising some restraint after a period of overextension. Nevertheless, because of its prevalence among the foreign policy executive now dominated by MbS, this perception significantly shaped choices, pushing foreign policy toward more assertiveness.

Acute perceptions of the threat of Iran similarly nudged Saudi Arabia toward overbalancing. There was at the time a deeply held belief among senior Saudi officials that Iran poses an existential threat. Most interviewees agreed that the Saudi leadership viewed the Islamic Republic as led by a messianic clan bent on imposing Persian and Shia domination. For example, MbS’s comparison of Iran’s Supreme Leader Ali Khamenei with Hitler^76^ is inaccurate (the Islamic Republic has neither the intentions nor the capabilities of Nazi Germany), but it reflects a common view at the time. As one Riyadh-based diplomat argued, ‘at the end of the day, it always boils to Iran when you talk to Saudi officials’.^77^ Saudi Arabia thus partly framed its intervention in Yemen as an effort to counter Iranian aggression. One Riyadh-based diplomat emphasized a point supported by multiple interviews: Saudi Arabia sees the Houthis as an arm of the Islamic Republic on its southern border.^78^

Saudi officials at the time overestimated the extent of Iranian support for, and influence over, the Houthis, an assessment shared by multiple interviewees among Riyadh-based foreign diplomats. Iran had been providing some support to the Houthis for a few years but, by March 2015, it was still marginal (though growing). Crucially, Iran’s limited support at the time did not allow it to exert much influence over Houthi decision-making or to make more than a marginal difference in the balance of forces inside Yemen.^79^ It is true that Tehran’s support for the Houthis has been rising since 2015 but, as will be discussed below, that is at least in part the result of the intervention itself.

In combination with the permissive structural context described above, Saudi Arabia perceived the aggregation of Iran-backed Houthi power on its vulnerable southern border as a significant threat. Acting as a complementary factor, this shaped the nature of the Saudi response. The second intervening variable, resource mobilization, acted as a moderating factor, shaping the intensity of the response.

Before 2015, the Saudi king was *primus inter pares* but would not make major decisions alone, or even with a close circle of advisors. Rather, he would build consensus among power centers within the royal family and the clergy, business elites, and security services. The resulting foreign policy was typically ponderous, cautious, and risk averse.^80^ As such, consolidation of power would represent an important facilitator of an overreaction as it would imply a greater ability to generate swift consensus among a smaller number of more like-minded elites. In other words, this intervening variable did not necessarily make an overbalancing reaction impossible prior to 2015, but it certainly made it less likely.

This is what occurred after early 2015. King Abdullah, on the throne since 2005, died in January and was succeeded by his half-brother Salman. The new king jettisoned the principle of horizontal succession among King Abdulaziz’s sons and restructured the line of succession by sidelining his half-brothers and naming as Crown Prince his nephew, the powerful Minister of the Interior Mohammed bin Nayef (MbN), and his son MbS as Deputy Crown Prince.^81^ Very quickly, power became increasingly centralized in the hands of MbS, who was named Minister of Defense, first chief of the royal court, and chair of the council of economic affairs. As early as March 2015, MbS had largely unchallenged control over defense, economic and oil policy; only the Interior Ministry (under MbN) and the National Guard (under one of Abdullah’s sons) remained outside his control. This trend continued until June 2017 when MbS formally became Crown Prince, sidelining MbN.^82^ This rapid consolidation of power was not complete when the intervention began in March 2015, but it was already well under way. In this context, there is no doubt that MbS was the ‘architect’ of the decision to launch the war in Yemen.^83^ Indeed, many senior officials, who would have been influential on such a decision prior to 2015, including MbN and senior royals close to Abdullah reportedly, opposed the decision, as confirmed by multiple interviews.

It might be tempting to assume that the first image explanation – the power of MbS’s personality and his worldview – overwhelms other variables and could, on its own, account for the overbalancing outcome. According to this line of criticism, a neoclassical realist framework is unnecessarily complex and heavy; a monocausal explanation should instead be sufficient. The leadership variable certainly has a strong explanatory role, but it is insufficient on its own. The independent variable, first, plays an easily neglected but essential role. A counterfactual can illustrate its importance. In the absence of the permissive causal role played by power – more specifically, the dyadic Saudi-Iranian competition and the power vacuum to Saudi Arabia’s south in 2015 – the incentives for Riyadh to commit so many resources would have been far weaker, while the constraints would have been stronger. Moreover, the third step – resource mobilization – is also necessary: in other circumstances with similar values on the independent and leadership images variables but a weak ability to mobilize resources, the state would be less likely to overbalance; it might even be impossible (and the response might be one of underbalancing) if the value of the resource mobilization variable were excessively weak. Put differently, the multiple steps in the causal chain are individually all necessary but not sufficient to explain the sub-optimal outcome; it is only taken together, in a comprehensive neoclassical realist explanation, that one can fully account for overbalancing.

### Dependent variable

By overbalancing instead of mounting a proportional balancing response against the threat emanating from Yemen, Saudi Arabia became stuck in a costly war.^84^ The gap between what should, ideally, have been a proportional balancing response, as prescribed by defensive realism, and reality, has been demonstrated by Rich and Moore-Gilbert: Saudi Arabia’s response to the situation in Yemen was not consistent with defensive realism, but was instead an ‘unprecedented expansive and interventionist behavior’.^85^ By mounting a balancing response, the balancer seeks to maximize its security; by overbalancing, Saudi Arabia failed to do so. This section demonstrates how this was the case, as Saudi Arabia failed to achieve its objectives of restoring the Hadi government and defeating the Houthis, and instead became bogged down in an unending quagmire.^86^

The war has damaged Saudi security. Most directly, the Houthis have lobbed hundreds of missiles and UAVs on Saudi territory, mostly in southwest Saudi Arabia, but in some cases deep inside the country. They have attacked Saudi pipelines and other critical infrastructure with missiles and drones; even if the economic and military damage has been limited, the Houthis have shown, and have openly signaled, that they have the capability and intent to make significantly more damage. They have also frequently launched raids into Saudi Arabia, sometimes occupying small areas.^87^ In 2021, they also started hitting Saudi maritime facilities by launching water-borne improvised explosive devices from ships in the Red Sea.^88^

The intervention has been costly financially. Estimates vary; interviewees in Riyadh proposed a range of 2–6 $billion per month at its height. The war, moreover, has been detrimental to Saudi efforts to attract foreign investment in the context of its program of economic reforms known as Vision 2030: foreign investment hit a 14-year low in 2017, with inflows shrinking from $7.5 billion in 2016 to $1.4 billion the next year.^89^

Interviewees also agreed that Saudi Arabia’s intervention has damaged its security by limiting its ability to gain from its most important partnerships, primarily with the main guarantor of its security. The United States provides Saudi Arabia with intelligence, air-to-air refueling, and technical assistance in its conduct of the war, and it has continued selling it weapons.^90^ Yet the intervention has damaged Saudi Arabia’s reputation in the United States (and beyond) because of the devastation wrought onto Yemen and its commitment of human rights abuses and war crimes.^91^ It has been widely criticized, in particular, for its indiscriminate airstrikes, which have killed thousands of civilians and destroyed much infrastructure in what was already the Arab world’s poorest country, and for its harsh blockade. This criticism has come from Western human rights organizations, but also from the media, some governments (e.g. Sweden, Germany), and, increasingly, the American Congress.

The United States has thus suffered costs by association, as have other Saudi partners in the West.^92^ This did not lead to a break in relations between Saudi Arabia and its Western partners. It did have consequences, however, as it constrained the willingness of the United States and other Western states to cooperate with the Kingdom. Especially in the wake of the murder of Jamal Khashoggi in 2018, growing media attention to arms sales to Saudi Arabia has been highly negative in North America and Europe, leading, for example, Germany to announce a suspension of arms sales and others to consider following suit.^93^ This represents another cost for Saudi Arabia and contributes to its failure to maximize its security: it restricted the United States’ willingness to cooperate, a loss for Riyadh as its ability to gain from its most important partnership was damaged.

Saudi Arabia’s overbalancing has, in addition, provoked a counter-reaction: its adversaries, the Houthis and Iran, counter-balanced against it more than they would have had Saudi Arabia acted proportionately. This is, again, costly. The war has benefitted the Houthis, the reverse outcome Saudi Arabia initially sought.^94^ On the one hand, it is true that the Houthis lost territory, especially in the south, in the early phase of the intervention. But the war has marginalized moderate voices within the movement and empowered hard-liners. The Houthis have since acquired significant amounts of light and heavy weapons by absorbing units previously loyal to Saleh, looting Yemeni military bases, developing alliances with tribal militias, and through Iranian assistance.^95^ The war has also allowed them to develop their fighting skills; they can now fight conventionally, which they had little experience doing before 2015. They have enhanced their already strong unconventional skills, notably in resisting aerial attacks and developed their ability to weld conventional and guerilla tactics.^96^

The war, moreover, has pushed the Houthis and Iran to significantly deepen their partnership. Iran’s presence in Yemen was limited in 2015 but has since increased; this, again, is precisely the reverse outcome Saudi Arabia sought.^97^ Iran, in particular, has been sending the Houthis larger amounts of light weapons and ammunitions, as well as more lethal and advanced equipment such as parts for drones and missiles. The UN Panel of Experts on Yemen has also documented how Iran started providing sea mines and anti-ship missiles, allowing the Houthis to project power into the Red Sea, an inconceivable outcome prior to 2015.^98^ Iran also provides training and advice on how to use these weapons, as well as intelligence.

The war has thus acted as a self-fulfilling prophecy: Saudi Arabia launched the intervention partly in response to an exaggerated perception of the Iranian threat, but its actions have amplified that threat, and has provided part of the impetus making it reality. It is plausible to estimate Iran’s support to the Houthis at a few tens of $millions, perhaps in the low hundreds per year. The war, on the other hand, cost Saudi Arabia $tens of billions annually in its first years. Iran is not solely responsible for the Saudis getting bogged down, but it has contributed to increasing the already high costs of Saudi overbalancing. This has led to a relative loss for Saudi Arabia: at a low cost, Iran has consolidated its partnership with the dominant force in Yemen and significantly raised costs for its Saudi rival by contributing to getting it bogged down. Iran has witnessed the Saudi military’s poor performance, its inability to seal its border against Houthi incursions, and its poor performance in intercepting Houthi missiles even though it possesses advanced air defense systems. More generally, Saudi Arabia’s poor military performance exposes its inability, despite its massive defense budget, to play the role of regional hegemon to which it aspires.

For Iran, overall, its involvement in Yemen has provided a good return on a limited investment: it has made important security gains by boosting its deterrent against Saudi Arabia and increasing its ability to impose costs on it. The result is the opposite for Saudi Arabia: despite a major investment in Yemen, it has suffered important damages to its security. This is textbook overbalancing: faced with a threat, Saudi Arabia failed to balance proportionately but instead generated a disproportionately strong amount of counterforce. In so doing, it has failed to maximize its security and has instead suffered important losses.

## Conclusion

A neoclassical theory of overbalancing performs well on multiple accounts. It has explanatory power, providing a nuanced and comprehensive account of the sub-optimal behavior of states overcommitting resources in response to a threat. It also has predictive value, as the likely evolution of sub-optimal behavior can be forecasted on the basis of the operation of the variables of its causal chain. And it has prescriptive value, in that it helps the overbalancer to identify the sources of its mistakes, and other states to identify pressure points and sources of leverage in their dealings with the overbalancer.

A neoclassical realist theory of overbalancing, first, provides a nuanced explanation of Saudi Arabia’s decision to launch its large-scale intervention in Yemen. It leads to an explanation superior to single-factor accounts. It sacrifices parsimony by incorporating multiple variables, but it gains accuracy and specificity. It is also comprehensive: it explains how the regional balance of power in the Arabian Peninsula and the Persian Gulf in combination with domestic political processes in Saudi Arabia steered the country toward costly overbalancing.

The theory of overbalancing has predictive value. By forecasting the likely evolution of its variables, it is possible to predict the evolution of Saudi assertiveness. MbS’s position, in particular, appears secure. One can thus expect that two elements explaining overbalancing – perceptions and the consolidation of power in his hands – will continue impacting Saudi foreign policy. Should MbS lose power, however, or should his worldview change, one would expect overbalancing to become significantly less likely.

Neoclassical realism is also useful for prescriptive purposes. By breaking down policymaking into a causal chain, it identifies pressure points for others seeking leverage. Evolving Saudi perceptions of the American regional role, in particular, modulate its assertiveness. Perceptions of abandonment drive assertiveness, but possibly also perceptions of blind support. In this sense, the optimal position for the United States may be to remain close to Saudi Arabia (it is better to have it as a partner, albeit a difficult one, than a rival), but not too close.

Counterfactuals can also illustrate the theory’s validity. The United States, for example, chose not to intervene on a large scale in Syria in 2012 and 2013, when such options were seriously considered by the Obama Administration. There is a strong defensive realist case to be made that this was the right decision: intervening in Syria’s brutal civil war would likely have led American forces to become bogged down in a costly intervention, with little prospect of success, in defense of what was a limited interest. What if the U.S. had intervened on a larger scale by deviating from the structurally-induced baseline – which prescribed that it should refrain from jumping into the Syrian war? A theory of overbalancing can explain how this scenario would have played out, and why it would have been costly. This represents a fruitful agenda for future research.

To be valid, finally, a theory should explain more than one puzzle. Further research would be necessary to validate this, but the theory of overbalancing seems generalizable to other cases. It could, for example, be applied to the case of American policy toward Iran: the Islamic Republic certainly poses a threat to U.S. interests, but the case can be made that Washington has overestimated this threat, leading it to overbalance. This has been costly: it has led Iran to escalate the dispute and to counterbalance, damaging the security of the U.S. and its regional partners, and it has forced the U.S. to expend considerable diplomatic capital, leading it to neglect other issues.

The next steps must also include the further theoretical development of the causal chain that leads to overbalancing. This article offered a first crack; subsequently, the independent, intervening, and dependent variables need to be refined, as would the more precise causal mechanisms linking them.

## Notes

1. The scope of this article is to study the decision itself, not the conduct of the war since.

2. Kenneth Waltz, *Theory of International Politics* (New York, NY: McGraw-Hill, 1979), pp. 156–7.

3. Joseph Parent and Sebastian Rosato, ‘Balancing in Neorealism’, *International Security*, 40(2), 2015, p. 56.

4. Kenneth Waltz, ‘Evaluating Theories’, *American Political Science Review*, 91(4), 1997, p. 915.

5. Randall Schweller, ‘Unanswered Threats: A Neoclassical Realist Theory of Underbalancing’, *International Security*, 29(2), 2004, p. 168.

6. F. Gregory Gause III, ‘Ideologies, Alignments, and Underbalancing in the New Middle East Cold War’, *PS: Political Science & Politics*, 50(3), 2017, pp. 672–5.

7. Schweller, ‘Unanswered Threats’, p. 167.

8. John Vasquez and Colin Elman (eds), *Realism and the Balance of Power: A New Debate* (Upper Saddle River, NJ: Prentice Hall, 2003); T. V. Paul, James Wirtz and Michel Fortmann (eds), *Balance of Power Theory: Theory and Practice in the 21*^st^ *Century* (Stanford, CA: Stanford University Press, 2004).

9. Stephen Walt, *The Origins of Alliances* (Ithaca, NY: Cornell University Press, 1987).

10. Steven David, ‘Explaining Third World Alignment’, *World Politics*, 43(2), 1991, pp. 233–56.

11. Robert Mason, *Foreign Policy in Iran and Saudi Arabia: Economics and Diplomacy in the Middle East* (London: I.B. Tauris, 2015); Gerd Nonneman, ‘Determinants and Patterns of Saudi Foreign Policy: ‘Omnibalancing’ and ‘Relative Autonomy’ in Multiple Environments’, in Paul Aarts and Gerd Nonneman (eds), *Saudi Arabia in the Balance: Political Economy, Society, Foreign Affairs* (New York, NY: New York University Press, 2005), pp. 315–51. On omnibalancing in the Middle East more broadly, see Raymond Hinnebusch, *The International Politics of the Middle East*, 2nd ed. (Manchester: Manchester University Press, 2015).

12. Jack Snyder, *Myths of Empire: Domestic Politics and International Ambition* (Ithaca, NY: Cornell University Press, 1991).

13. Schweller, ‘Unanswered Threats’, p. 182.

14. Robert Jervis, *Perception and Misperception in International Politics* (Princeton, NJ: Princeton University Press, 1976).

15. William Wohlforth, *The Elusive Balance: Power and Perceptions during the Cold War* (Ithaca, NY: Cornell University Press, 1993).

16. This article offers a first crack at a neoclassical realist theory of overbalancing, and then applies it to the Saudi case. For a more in-depth effort at theorizing overbalancing, see Ellis Mallett and Thomas Juneau, ‘A Neoclassical Realist Theory of Overbalancing’, *Global Studies Quarterly*, 3(2), 2023, pp. 1–12.

17. Gideon Rose, ‘Neoclassical Realism and Theories of Foreign Policy’, *World Politics*, 51(1), 1998, pp. 144–72.

18. Norrin Ripsman, Jeffrey Taliaferro and Steven Lobell, *Neoclassical Realist Theory of International Politics* (Oxford: Oxford University Press, 2016).

19. Brian Rathbun, ‘A Rose by Any Other Name: Neoclassical Realism as the Logical and Necessary Extension of Structural Realism’, *Security Studies*, 17(2), 2008, pp. 294–321.

20. Ripsman et al., *Neoclassical Realist Theory of International Politics.*

21. For more on how Type I neoclassical realism can be operationalized, see Thomas Juneau, ‘Neoclassical Realism as a Theory for Correcting Mistakes: What State X Should Do Next Tuesday’, *International Studies Review*, 25(3), 2023, viad021, DOI: 10.1093/isr/viad021.

22. Steven Lobell, ‘A Granular Theory of Balancing’, *International Studies Quarterly*, 62(3), 2018, pp. 593–605.

23. Brian Schmidt and Thomas Juneau, ‘Neoclassical Realism and Power’, in Asle Toje and Barbara Kunz (eds), *Neoclassical Realism in European Politics: Bringing Power Back In* (Manchester: Manchester University Press), pp. 61–78.

24. Barry Posen, *Restraint: A New Foundation for U.S. Grand Strategy* (Ithaca, NY: Cornell University Press, 2014).

25. Stephen Walt, ‘The Case for Finite Containment: Analyzing U.S. Grand Strategy’, *International Security*, 14(1), 1989, pp. 5–49.

26. Steven Lobell, Norrin Ripsman and Jeffrey Taliaferro (eds), *Neoclassical Realism, the State, and Foreign Policy* (Cambridge: Cambridge University Press, 2009).

27. Thomas Juneau, *Squandered Opportunity: Neoclassical Realism and Iranian Foreign Policy* (Stanford, CA: Stanford University Press, 2015), p. 24.

28. Hans Morgenthau, *Politics Among Nations: The Struggle for Power and Peace*, 6th ed. (Boston, MA: McGraw Hill, 1993), p. 7.

29. Kenneth Waltz, *Man, the State, and War: A Theoretical Analysis* (New York, NY: Columbia University Press, 1959), p. 238.

30. Juneau, *Squandered Opportunity*, p. 25.

31. Nicholas Kitchen, ‘Systemic Pressures and Domestic Ideas: A Neoclassical Realist Model of Grand Strategy Formulation’, *Review of International Studies*, 36(1), 2010, pp. 117–43.

32. Jeffrey Legro and Andrew Moravcsik, ‘Is Anybody Still a Realist?’, *International Security*, 24(2), 1999, pp. 5–55; Kevin Narizny, ‘On Systemic Paradigms and Domestic Politics: A Critique of the Newest Realism’, *International Security*, 42(2), 2017, pp. 155–90.

33. For a stock-taking exercise, see Gustav Meibauer, Linde Desmaele, Tudor Onea, et al., ‘Forum: Rethinking Neoclassical Realism at Theory’s End’, *International Studies Review*, 23(1), 2021, pp. 268–95.

34. Michiel Foulon, ‘Neoclassical Realist Analyses of Foreign Policy’, Oxford Research Encyclopedia of Politics, 2017, DOI: 10.1093/acrefore/9780190228637.013.387.

35. Ripsman et al., *Neoclassical Realist Theory of International Politics*, pp. 59–79.

36. Elias Götz, ‘Neoclassical Realist Theories, Intervening Variables, and Paradigmatic Boundaries’, *Foreign Policy Analysis*, 17(2), 2021, p. 5.

37. Götz, ‘Neoclassical Realist Theories’, p. 3.

38. Jervis, *Perception and Misperception*.

39. Jeffrey Taliaferro, ‘Security Seeking Under Anarchy: Defensive Realism Revisited’, *International Security*, 25(3), 2000/01, pp. 128–61.

40. Gustav Meibauer, ‘Interests, Ideas, and the Study of State Behavior in Neoclassical Realism’, *Review of International Studies*, 46(1), 2020, pp. 20–36.

41. Ripsman et al., *Neoclassical Realist Theory of International Politics*, p. 61.

42. Daniel Byman and Kenneth Pollack, ‘Let Us Now Praise Great Men: Bringing the Statesman Back In,’ *International Security*, 25(4), 2001, pp. 107–46.

43. Wohlforth, *The Elusive Balance*; Kei He, ‘Explaining United States-China Relations: Neoclassical Realism and the Nexus of Threat-Interest Perceptions’, *The Pacific Review*, 30(2), 2017, pp. 133–51.

44. Schweller, ‘Unanswered Threats’.

45. Jeffrey Taliaferro, ‘State Building for Future War: Neoclassical Realism and the Resource Extractive State’, *Security Studies*, 15(3), 2006, pp. 464–95.

46. Fareed Zakaria, *From Wealth to Power: The Unusual Origins of America’s World Role* (Princeton, NJ: Princeton University Press, 1998).

47. Ripsman et al., *Neoclassical Realist Theory of International Politics*, pp. 99–138.

48. This section is partly built on about 40 interviews conducted in 2018 and 2019 in Saudi Arabia (Riyadh and Jeddah) and the United Arab Emirates (Abu Dhabi) with Saudi and Emirati officials, exiled Yemeni officials, local and foreign academics, foreign diplomats, journalists, and activists. All were granted anonymity. These interviews were especially central to identify and validate the relevance of the two intervening variables and to gain insight on the details of their operationalization.

49. Joseph Mann, ‘Yemeni Threat to Saudi Arabia’s Internal Security, 1962-1970’, *Journal of Arabian Studies*, 4(1), 2014, pp. 52–69.

50. Ginny Hill, *Yemen Endures: Civil War, Saudi Adventurism, and the Future of Arabia* (Oxford: Oxford University Press, 2017).

51. Noel Brehony, *Yemen Divided: The Story of a Failed State in South Arabia* (London: I.B. Tauris, 2011).

52. Marieke Brandt, *Tribes and Politics in Yemen: A History of the Houthi Conflict* (London: Hurst, 2017).

53. Marie-Christine Heinze (ed.), *Yemen and the Search for Stability: Power, Politics and Society after the Arab Spring* (London: I.B. Tauris, 2018).

54. Karim Umer, ‘The Evolution of Saudi Foreign Policy and the Role of Decision-Making Processes and Actors’, *The International Spectator*, 52(2), 2017, pp. 71–88; Ben Rich and Kylie Moore-Gilbert, ‘From Defense to Offense: Realist Shifts in Saudi Foreign Policy’, *Middle East Policy*, 26(3), 2019, pp. 62–76; Tyler Parker, ‘Transforming Yemen? Divergent Saudi and Emirati Intervention Policies’, *Middle East Policy*, 28(3–4), 2021, pp. 159–71.

55. Christopher Davidson, *From Sheikhs to Sultanism: Statecraft and Authority in Saudi Arabia and the UAE* (Oxford: Oxford University Press, 2021).

56. Madawi Al-Rasheed, ‘Brute Force and Hollow Reforms in Saudi Arabia’, *Current History*, 119(821), 2020, pp. 331–37.

57. May Darwich, ‘The Saudi Intervention in Yemen: Struggling for Status’, *Insight Turkey*, 20(2), 2018, pp. 125–41.

58. May Darwich, ‘Escalation in Failed Military Interventions: Saudi and Emirati Quagmires in Yemen’, *Global Policy*, 11(1), 2020, pp. 103–12.

59. Oz Hassan, ‘Undermining the Transatlantic Democracy Agenda? The Arab Spring and Saudi Arabia’s Counteracting Democracy Strategy’, *Democratization*, 22(3), 2015, pp. 479–95.

60. Eman Ragab, ‘Beyond Money and Diplomacy: Regional Policies of Saudi Arabia and UAE after the Arab Spring’, *The International Spectator*, 52(2), 2017, pp. 37–53.

61. Asher Orkaby, ‘Yemen’s Humanitarian Nightmare: The Real Roots of the Conflict’, *Foreign Affairs*, 96(6), 2017, pp. 93–101.

62. F. Gregory Gause III, ‘Policy Roundtable 2-4: The Saudi-Iranian Cold War’, H-Diplo/ISSF, 20 February 2018, available at: https://issforum.org/roundtables/policy/2-4-saudi-iranian (accessed 22 March 2021).

63. Nader Hashemi and Danny Postel (eds), *Sectarianization: Mapping the New Politics of the Middle East* (London: Hurst, 2017); for background, see Toby Matthiesen, *Sectarian Gulf: Bahrain, Saudi Arabia, and the Arab Spring that Wasn’t* (Stanford, CA: Stanford University Press, 2013).

64. Madawi Al-Rasheed, ‘Sectarianism as Counter-Revolution: Saudi Responses to the Arab Spring’, *Studies in Ethnicity and Nationalism*, 11(3), 2011, pp. 513–26.

65. Thomas Demmelhuber, ‘Playing the Diversity Card: Saudi Arabia’s Foreign Policy under the Salmans’, *The International Spectator*, 54(4), 2019, pp. 109–24. See also Anna Jacobs, ‘Understanding Saudi Arabia’s Recalibrated Foreign Policy’, *International Crisis Group*, 14 September 2023, available at: https://www.crisisgroup.org/middle-east-north-africa/gulf-and-arabian-peninsula/saudi-arabia/understanding-saudi-arabias (accessed 17 October 2023).

66. Vincent Durac, ‘The Limits of the Sectarian Narrative in Yemen’, *Global Discourse*, 9(4), 2019, pp. 655–73.

67. Anoushiravan Ehteshami, ‘Saudi Arabia as a Resurgent Regional Power’, *The International Spectator*, 53(4), 2018, pp. 75–94.

68. Anna Sunik, ‘Regional Leadership in Authoritarian Contexts – Saudi Arabia’s New Military Interventionism as Part of its Leadership Bid in the Middle East’, *Rising Powers Quarterly*, 3(1), 2018, pp. 65–85.

69. Thomas Juneau, ‘Iran’s Policy Towards the Houthis in Yemen: A Limited Return on a Modest Investment’, *International Affairs*, 92(3), 2016, pp. 647–63.

70. Simon Mabon, *Saudi Arabia and Iran: Power and Rivalry in the Middle East* (London: I.B. Tauris, 2016).

71. Thomas Juneau, ‘Iran Under Rouhani: Still Alone in the World’, *Middle East Policy*, 21(4), 2014, pp. 92–104.

72. Thomas Juneau, ‘How War in Yemen Transformed the Iran-Houthi Partnership’, *Studies in Conflict and Terrorism*, 2021, available at: https://www.tandfonline.com/doi/full/10.1080/1057610X.2021.1954353 (accessed 2 March 2022).

73. Quoted in Patrick Cockburn, ‘Prince Mohammed bin Salman: Naive, Arrogant Saudi Prince Is Playing with Fire’, *The Independent*, 9 January 2016, available at: https://www.independent.co.uk/news/world/middle-east/prince-mohammed-bin-salman-naive-arrogant-saudi-prince-is-playing-with-fire-a6804481.html (accessed 16 May 2018).

74. Christopher Philipps, ‘Eyes Bigger than Stomachs: Turkey, Saudi Arabia and Qatar in Syria’, *Middle East Policy*, 24(1), 2017, pp. 36–47.

75. A. K. Pasha, ‘Saudi Arabia and the Iranian Nuclear Deal’, *Contemporary Review of Middle East Studies*, 3(4), 2016, pp. 387–404. This perception was evident in interviews with Saudi officials and analysts, and foreign diplomats, in Riyadh.

76. Jeffrey Goldberg, ‘Saudi Crown Prince: Iran’s Supreme Leader Makes Hitler Look Good’, *The Atlantic*, 2 April 2018.

77. For a semi-official expression of this view, see the November-December 2017 issue of *The Diplomat*, a magazine published by the Prince Saud Al Faisal Institute for Diplomatic Studies, the Saudi Ministry of Foreign Affairs think-tank, “Mullahs’ Missiles Threaten World Peace”.

78. Illustrating the prevalence of this view, a Saudi human rights activist, in an interview, was severely critical of Saudi Arabia’s domestic policies and of much of its foreign policy – but was convinced of the need to aggressively counter Iran.

79. Juneau, ‘Iran’s Policy Towards the Houthis’.

80. Madawi Al-Rasheed (ed.), *Salman’s Legacy: The Dilemmas of a New Era in Saudi Arabia* (London: Hurst, 2018).

81. Madawi Al-Rasheed, *The Son King: Reform and Repression in Saudi Arabia* (London: Hurst, 2021).

82. F. Gregory Gause III, ‘Fresh Prince: The Schemes and Dreams of Saudi Arabia’s Next King’, *Foreign Affairs*, 97(3), 2018, pp. 75–86.

83. Bruce Riedel, ‘The Man Who Would Be King in Saudi Arabia’, Brookings Institution, 10 May 2016, available at: https://www.brookings.edu/opinions/the-man-who-would-be-king-in-saudi-arabia/ (accessed 28 April 2018).

84. Ibrahim Jalal, ‘Saudi Arabia Eyes the Exit in Yemen, but Saudi-Houthi Talks Alone Won’t Resolve the Conflict’, Middle East Institute, 15 April 2020, available at: https://www.mei.edu/publications/saudi-arabia-eyes-exit-yemen-saudi-houthi-talks-alone-wont-resolve-conflict (accessed 12 December 2020).

85. Rich and Moore-Gilbert, ‘From Defense to Offense’, p. 70.

86. Sana’a Center, ‘The Graveyard of Hubris – Yemen Annual Review 2021’, 3 March 2022, available at: https://sanaacenter.org/publications/the-yemen-review/16768 (accessed 4 March 2022).

87. Seth G. Jones, Jared Thompson, Danielle Ngo, et al., ‘The Iranian and Houthi War against Saudi Arabia’, CSIS Briefs, December 2021, available at: https://csis-website-prod.s3.amazonaws.com/s3fs-public/publication/211221_Jones_IranianHouthi_SaudiArabia.pdf?fn1d98tAhj7yOUr.IncppMueLOC4kv83 (accessed 21 January 2022).

88. UN Panel of Experts on Yemen, ‘Report 2021’, 22 January 2022, available at: https://www.securitycouncilreport.org/atf/cf/%7B65BFCF9B-6D27-4E9C-8CD3-CF6E4FF96FF9%7D/S_2022_50.pdf (accessed 27 January 2022).

89. Gulf Business, ‘Foreign Investment in Saudi at 14-Year low Despite Reforms’, 10 June 2018, available at: http://gulfbusiness.com/foreign-investment-saudi-14-year-low-despite-reforms/ (accessed 13 October 2018).

90. Ziad Al-Eryani and Holly Topham, ‘The US in Yemen: Facilitating Disaster, Dodging Culpability’, Sana’a Center, 9 April 2020, available at: https://sanaacenter.org/publications/analysis/9605 (accessed 15 April 2020).

91. Multiple human rights organizations have written on Saudi war crimes in Yemen (and noted that other parties to the conflict also committed such violations); Amnesty International, ‘Human Rights in Yemen: Review of 2018’, 26 February 2019, available at: https://www.amnesty.org/en/documents/mde31/9898/2019/en/ (accessed 13 March 2020).

92. Daniel Byman, ‘The U.S. ‘Yellow Light’ in Yemen’, Lawfare, 3 August 2018, available at: https://www.lawfareblog.com/us-yellow-light-yemen (accessed 29 August 2018).

93. Rick Noack, ‘Germany Halts Arms Deals with Saudi Arabia, Encourages Allies to Do the Same’, The Washington Post, 22 October 2018, available at: https://www.washingtonpost.com/world/2018/10/22/germany-its-allies-well-halt-future-arms-sales-saudi-arabia-until-we-have-clarity-khashoggi-so-should-you/ (accessed 27 February 2022). Western diplomats interviewed in Riyadh and Abu Dhabi all agreed with the proposition that the war in Yemen did not make cooperation with Saudi Arabia impossible, but did raise its price.

94. Ibrahim Jalal, ‘Five Years on, Has the Arab Coalition Achieved its Objectives in Yemen?’, Middle East Institute, 2 April 2020, available at: https://www.mei.edu/publications/five-years-has-arab-coalition-achieved-its-objectives-yemen (accessed 17 April 2020).

95. Jean-Loup Samaan, ‘Missiles, Drones, and the Houthis in Yemen’, *Parameters*, 50(1), 2020, 51–64.

96. Michael Knights, ‘Yemen’s Southern Hezbollah: Implications of Houthi Missile and Drone Improvements’, Washington Institute for Near East Policy, 1 April 2021, available at: https://www.washingtoninstitute.org/policy-analysis/yemens-southern-hezbollah-implications-houthi-missile-and-drone-improvements (accessed 23 April 2021).

97. Juneau, ‘How War in Yemen Transformed the Iran-Houthi Partnership’.

98. See for example its 2019 report, p. 83. UN Panel of Experts on Yemen, ‘2019 Report’, available at: https://www.securitycouncilreport.org/atf/cf/%7B65BFCF9B-6D27-4E9C-8CD3-CF6E4FF96FF9%7D/S_2020_70.pdf (accessed 21 February 2022).

